# Association mapping across a multitude of traits collected in diverse environments in maize

**DOI:** 10.1093/gigascience/giac080

**Published:** 2022-08-23

**Authors:** Ravi V Mural, Guangchao Sun, Marcin Grzybowski, Michael C Tross, Hongyu Jin, Christine Smith, Linsey Newton, Carson M Andorf, Margaret R Woodhouse, Addie M Thompson, Brandi Sigmon, James C Schnable

**Affiliations:** Center for Plant Science Innovation, University of Nebraska–Lincoln, Lincoln, NE 68588, USA; Department of Agronomy and Horticulture, University of Nebraska–Lincoln, Lincoln, NE 68588, USA; Center for Plant Science Innovation, University of Nebraska–Lincoln, Lincoln, NE 68588, USA; Department of Agronomy and Horticulture, University of Nebraska–Lincoln, Lincoln, NE 68588, USA; Center for Plant Science Innovation, University of Nebraska–Lincoln, Lincoln, NE 68588, USA; Department of Agronomy and Horticulture, University of Nebraska–Lincoln, Lincoln, NE 68588, USA; Center for Plant Science Innovation, University of Nebraska–Lincoln, Lincoln, NE 68588, USA; Department of Agronomy and Horticulture, University of Nebraska–Lincoln, Lincoln, NE 68588, USA; Center for Plant Science Innovation, University of Nebraska–Lincoln, Lincoln, NE 68588, USA; Department of Agronomy and Horticulture, University of Nebraska–Lincoln, Lincoln, NE 68588, USA; Center for Plant Science Innovation, University of Nebraska–Lincoln, Lincoln, NE 68588, USA; Department of Plant Soil and Microbial Sciences, Michigan State University, East Lansing, MI 48824, USA; USDA-ARS, Corn Insects and Crop Genetics Research Unit, Ames, IA 50010, USA; Department of Computer Science, Iowa State University, Ames, IA 50011, USA; USDA-ARS, Corn Insects and Crop Genetics Research Unit, Ames, IA 50010, USA; Department of Plant Soil and Microbial Sciences, Michigan State University, East Lansing, MI 48824, USA; Department of Plant Pathology, University of Nebraska–Lincoln, Lincoln, NE 68588, USA; Center for Plant Science Innovation, University of Nebraska–Lincoln, Lincoln, NE 68588, USA; Department of Agronomy and Horticulture, University of Nebraska–Lincoln, Lincoln, NE 68588, USA

**Keywords:** quantitative genetics, community association populations, pleiotropy, maize

## Abstract

Classical genetic studies have identified many cases of pleiotropy where mutations in individual genes alter many different phenotypes. Quantitative genetic studies of natural genetic variants frequently examine one or a few traits, limiting their potential to identify pleiotropic effects of natural genetic variants. Widely adopted community association panels have been employed by plant genetics communities to study the genetic basis of naturally occurring phenotypic variation in a wide range of traits. High-density genetic marker data—18M markers—from 2 partially overlapping maize association panels comprising 1,014 unique genotypes grown in field trials across at least 7 US states and scored for 162 distinct trait data sets enabled the identification of of 2,154 suggestive marker-trait associations and 697 confident associations in the maize genome using a resampling-based genome-wide association strategy. The precision of individual marker-trait associations was estimated to be 3 genes based on a reference set of genes with known phenotypes. Examples were observed of both genetic loci associated with variation in diverse traits (e.g., above-ground and below-ground traits), as well as individual loci associated with the same or similar traits across diverse environments. Many significant signals are located near genes whose functions were previously entirely unknown or estimated purely via functional data on homologs. This study demonstrates the potential of mining community association panel data using new higher-density genetic marker sets combined with resampling-based genome-wide association tests to develop testable hypotheses about gene functions, identify potential pleiotropic effects of natural genetic variants, and study genotype-by-environment interaction.

## Introduction

Association mapping, initially on a gene-by-gene level and later at a genome-wide scale, has been widely adopted as a tool to identify natural genetic variants controlling variation in both quantitative and qualitative traits. In the plant genetics community, logistical and scientific constraints have driven the development and widespread adoption of community association panels comprising sets of distinct plant genotypes that can be propagated and shared, whether through the use of homozygous inbred lines or clonal propagation. In the earlier era where association mapping was conducted on a gene-by-gene level, the use of community association panels allowed the work of estimating population structure within the population to be conducted once rather than for each independent study. In the later era of genome-wide association studies, the use of community association panels again provided substantial practical benefits: the time-consuming and expensive process of genotyping hundreds of thousands or millions of genetic markers across hundreds of individuals had to be undertaken only once to enable an effectively infinite number of studies on the genes controlling different traits by different research groups.

The use of community association panels by many independent research groups to investigate diverse research questions results in data on a wide range of individual traits for genetically identical individuals across 1 or more environments. This provides significant opportunities to investigate both pleiotropy, the effect of a single genetic locus on multiple phenotypes, and genotype-by-environment interactions, where the same allele influences the same phenotype in different ways in different environments. In addition, associations between a given genetic locus and a particular trait that are only marginally significant in several individual studies can often be assigned a higher degree of confidence when the same association is identified across multiple studies. Finally, marginal statistical signals from genome-wide association studies can assist in the interpretation of later mutant mapping, gene expression, or selection scans, but only if the initial studies results are made available in a method that is easy to capture and cross-reference.

In maize, an early widely adopted community association panel was the Maize Association Panel (MAP), also referred to variously as the maize 282 panel and the Buckler–Goodman Association panel (Supplementary Table S1). MAP initially consisted of 302 diverse inbreds estimated to represent 80% of the genetic diversity within maize, although several of these were dropped in later years based on poor seed increasability or other factors [1, 2]. Slow decreases in population size over time are a common feature of many community association panels. Two challenges were observed with the initial maize association panel. First, while the MAP panel captured a large proportion of total maize genetic diversity, it consisted primarily of older public-sector lines with limited representation of current temperate elite germplasm. Second, many of the MAP lines were difficult to grow and increase in the northern US corn belt. As a result, 2 additional panels were generated for use in the temperate United States: (i) the Shoot Apical Meristem association panel (SAM panel), which included many of the MAP genotypes augmented with expired Plant Variety Patent lines generated by the major seed companies in the United States [3], and (ii) the Wisconsin Diversity Panel (WiDiv), developed by selecting nonredundant and diverse genotypes that were able to complete their life cycle and produce significant amounts of seed when grown in Madison, Wisconsin [4]. In parallel, region-specific community association panels have been developed in other major corn-producing regions of the world, including the AM508 panel incorporating lines from CIMMYT and both tropical and temperate maize breeding programs in China [5] and the CornFed panel assembled with the goal of representing the genetic diversity in the flint and dent heterotic groups widely employed for hybrid maize production in Europe [6].

As sequencing technologies have improved, new sets of genetic markers have been deployed for existing community association panels with increasing degrees of genetic resolution. The MAP was initially genotyped with a modest number of (<100) Simple Sequence Repeat (SSR) markers to estimate population structure as a potential confounder for single-gene association tests [2]. The WiDiv panel was initially genotyped with 1,536 microarray-based markers [4]. The SAM panel was initially genotyped using sequencing of messenger RNA (mRNA) samples from each line, enabling the identification and scoring of 1.2M segregating single-nucleotide polymorphism (SNP) markers [3]. A new high-density marker set for the WiDiv panel was also generated by sequencing mRNA samples, providing a set of 900k segregating genetic markers in this population [7,8]. The original MAP population, which shares many genotypes with both the SAM and WiDiv panels, was resequenced as part of the Maize HapMap3 project, increasing the number of segregating genetic markers to 83M [9]. A subset of lines from the WiDiv panel was resequenced, resulting in a set of 3.1M SNPs scored across 511 genotypes [10].

Substantial barriers to the comparison, reuse, and meta-analysis of previously published genome-wide association study (GWAS) results are created by differences in genetic marker data sets as well as the use of different reference genome versions. In this study, we sought to generate a single common genetic marker set spanning multiple association panels with a high marker density. Specifically, we employed a combination of published RNA sequencing (RNA-seq) and resequencing data to generate a common set of  18M genetic markers scored across the union of 1,014 genotypes present in the SAM and/or WiDiv association panels. Given the expected complexity of the genetic architectures controlling many quantitative traits in maize, we employed the Fixed and random model Circulating Probability Unification (FarmCPU) algorithm for genome-wide association [11]. While the FarmCPU algorithm provides greater power to detect true-positive signals, the set of positive associated signals identified by the algorithm can vary significantly based on moderately sized changes in the composition of the studied population [12, 13]. The relative stability of GWAS signals identified via the FarmCPU algorithm can be assessed by evaluating the resample model inclusion probability (RMIP) of individual signals across multiple bootstraps [14], and this approach has been employed by a number of research groups working in different crop species [13,15, 16]. Resampling provides greater confidence in associations by quantifying the stability of signals, but also provides the opportunity to identify comparatively strong signals (e.g., those identified in 20–50% of total bootstraps), which will frequently be missed in a single analysis of the entire data set. We assemble a set of 162 trait data sets that have been scored across different subsets of these 1,014 genotypes, including both previously published studies conducted across seve7states and new trait data collected from field trials conducted in Lincoln, Nebraska. We employ these trait data, combining the genetic marker data set and genome-wide association approach described above, to evaluate both mapping resolution—the distance between significant marker trait associations and known or likely causal genes—and the incidence and patterns of quantitative pleiotropy across related and dissimilar traits. In this study, we generate and release data on genomic intervals containing 2,154 confident or suggestive marker trait associations across these 162 trait data sets to aid in the reuse of these trait data in future genomic and genetic studies, including suggestive signatures of pleiotropic effects for a number of genetic loci.

## Results

### Properties of widely studied maize association panels

**Figure 1:**
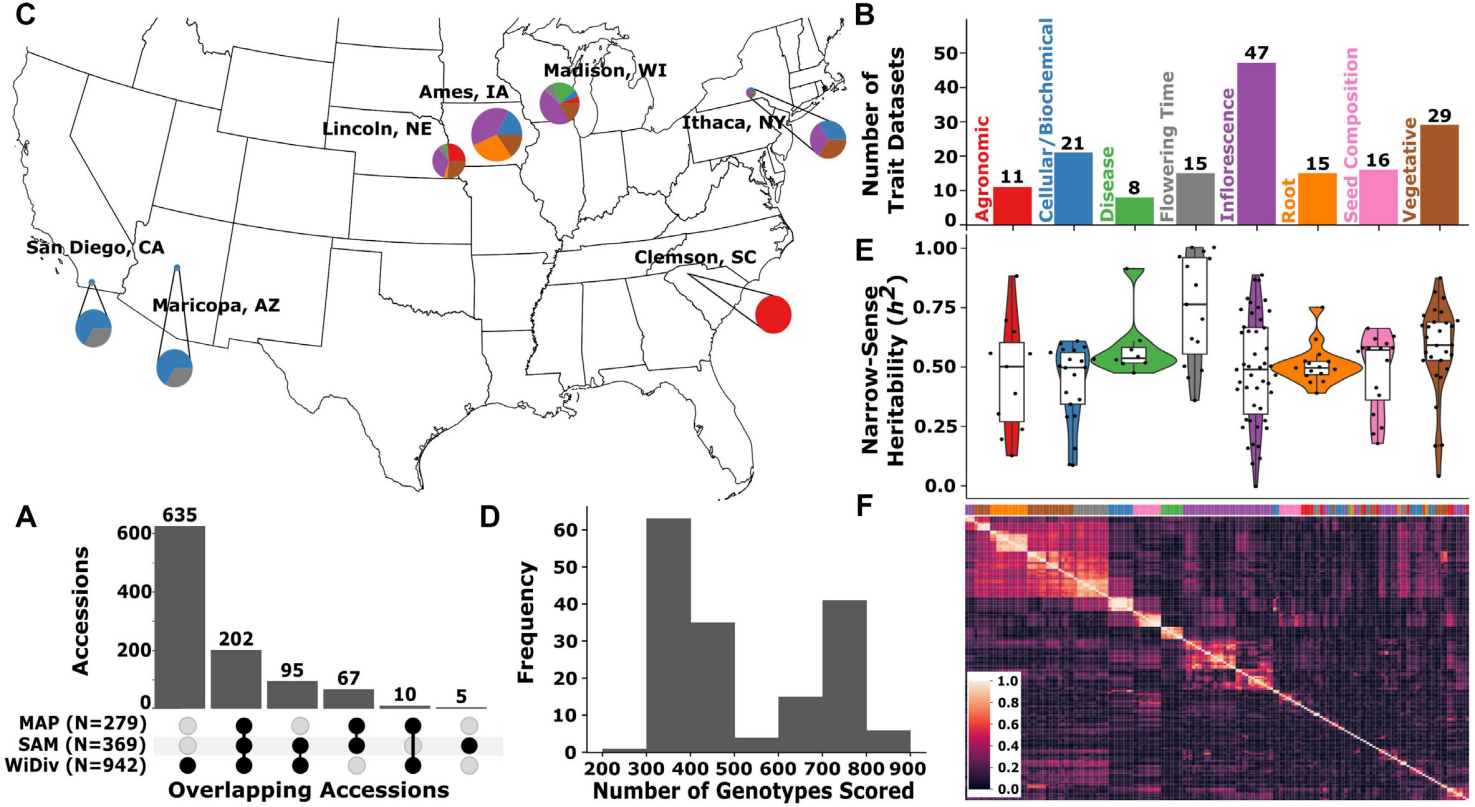
Characteristics of Maize Association Panel trait data sets. (A) Number of accessions that are represented in any of the 3 diversity panels. (B) Representation of 8 broad phenotypic categories among the 162 traits collected here. Category assignments for individual traits are provided in Supplementary Table S3. (C) Geographic distribution of trials where trait data sets were collected. Size of circles indicates number of traits collected at a specific geographic location. Colors of circles indicate types of trait data sets collected at that location. Labels for which colors correspond to which types of traits are given in panel B. (D) Distribution of the number of genotypes scored for a given trait. (E) Distributions of narrow-sense heritability values, across the same 8 broad phenotypic categories shown in panel B. Colors corresponding to the color key for phenotype classes are provided in panel B. (F) Correlations among the 162 trait data sets analyzed in this study. Trait data sets are clustered based upon absolute Spearman correlation value. Phenotype classes are indicated with color bar on top the x-axis with colors corresponding to the color key for phenotype classes provided in panel B.

Three maize association panels were identified in the literature: the Maize Association Panel (MAP) [2], the Shoot Apical Meristem (SAM) panel (369 lines) [3], and the Wisconsin Diversity (WiDiv) panel consisting of either 627 or 942 lines [4,8]. The latter 2 populations are largely supersets of MAP, with SAM excluding 10 MAP lines present in the WiDiv panel and WiDiv excluding 67 lines retained in both the SAM and MAP populations (Fig. 1A). The SAM and WiDiv populations also share 95 lines not present in the MAP population that served as a partial progenitor for both. These are predominantly more recently released lines developed in the private plant breeding sector and released when the associated plant variety parents expired (expired Plant Variety Patents or exPVPs). The total overlap between the SAM and WiDiv populations was 297, sufficient to enable joint analyses of trait data sets collected in these 2 populations. The union of the SAM and WiDiv populations included 1,014 unique maize genotypes with both genetic marker information for at least 1 phenotypic record. An additional 35 unique maize genotypes were included in 1 or both populations, had at least 1 source of genetic marker data but no phenotypic records, and so were excluded from downstream analyses.

The set of approximately 200 papers citing either the SAM panel [3] or either iteration of the WiDiv panel [4,8] was screened to identify studies that conducted GWAS and published trait data sets collected from 1 or both of these populations. A total of 21 papers were identified that included GWAS results generated from these populations. After excluding studies where we were unable to locate trait data for individual maize lines, excluding traits that failed initial quality control, and condensing studies that utilized previously published data, 133 unique trait data sets drawn from 16 separate published studies remained (Table 1). This included 55 trait data sets collected from the SAM panel, 67 trait data sets collected from the WiDiv panel, and 11 trait data sets collected from an even larger population of 2,815 maize lines with substantial overlap with these 2 populations [17]. An additional 29 phenotypes scored in Lincoln, Nebraska, in 2020 were included for a final set of 162 trait data sets employed for downstream analyses (Supplementary Table S3). Individual trait data sets included data values for between 222 and 817 maize lines (Fig. 1B) and were collected from field or controlled environment studies conducted in 7 states (Fig. 1C). Measurements related to inflorescence architecture were the most abundant category among these 162 trait data sets (Fig. 1D). SNP-based estimates of narrow-sense heritability for individual trait data sets were variable, with a median value of 0.527 and a mean value of 0.523 across all traits. Traits related to flowering time (e.g., timing of anthesis, timing of silking, or anthesis-silking interval) was the category that exhibited the highest median heritability of 0.762 (Fig. 1E and Supplementary Table S3). Flowering time traits collected in different environments were correlated with each other and also exhibited notable correlations with a subset of both above-ground vegetative and below-ground root-related traits (Fig. 1F).

**Table 1:** Studies from which maize trait data sets were drawn

Reference	Study type	Phenotypes scored^a^	Accessions evaluated^b^	Panel
Peiffer et al. 2014 [23]	Reproductive & Vegetative	11	737	Ames Panel
Hirsch et al. 2014 [7]	Reproductive & Vegetative	3	427	WiDiv-503
Leiboff et al. 2015 [3]	Agronomic, Cellular/Biochemical, & Vegetative	9	378	SAM
Lin et al. 2017 [24]	Cellular/Biochemical, Root, & Vegetative	16	363	SAM
Gustafson et al. 2018 [25]	Disease	7	447	WiDiv-503
Gage et al. 2018 [26]	Reproductive	16	817	WiDiv-942
Mazaheri et al. 2019 [8]	Cellular/Biochemical & Vegetative	5	788	WiDiv-942
Qiao et al. 2019 [27]	Cellular/Biochemical	4	429	WiDiv-503
Sekhon et al. 2019 [28]	Agronomic	3	364	WiDiv-503
Zheng et al. 2019 [29]	Agronomic, Root	13	359	SAM
Azodi et al. 2020 [30]	Reproductive & Vegetative	3	388	WiDiv-503
Lin et al. 2020 [31]	Cellular/Biochemical & Reproductive	8	439	WiDiv-503
Renk et al. 2021 [32]	Seed Composition	16	499	WiDiv-503
Schneider et al. 2021 [33]	Root	1	599	WiDiv-503
Zhou et al. 2021 [34]	Reproductive	17	339	SAM
Sun et al. 2021 [13]	Disease	1	687	WiDiv-942
Previously unpublished	Agronomic, Disease, Reproductive, & Vegetative	29	752	WiDiv-942

^a^Phenotypes used in this study from the phenotypes scored in respective studies (after removing exact same phenotype values if used in another study).

^b^The highest number of accessions with phenotype data used in this study from the respective publication.

The total number of unique maize line names observed across these 162 trait data sets was 1,118, which was modestly more than the set of 1,014 unique genotypes present across the WiDiv and SAM mapping populations. We speculate that this difference may result from the inclusion of local checks or lines of interest or changes in naming convention or transcription errors that we were unable to resolve. For the 1,014 unique genotypes named as part of the SAM population [3] or WiDiv population [8], raw whole-genome sequencing or RNA-seq sequence data were aggregated from a number of sources (Supplementary Table S2) [8, 9, 18] as described in Sun et al. [13]. Alignment of published sequence data from these sources to the maize reference genome (B73_RefGen_V4) [19,20], scoring of *a priori* segregating SNPs from HapMap3 [9], imputation, and filtering resulted in a set of 17,717,568 with minor allele frequency >0.01 and heterozygosity rate of <0.1, leading to an average of 1 SNP per 120 bp (see Materials and Methods).

**Figure 2:**
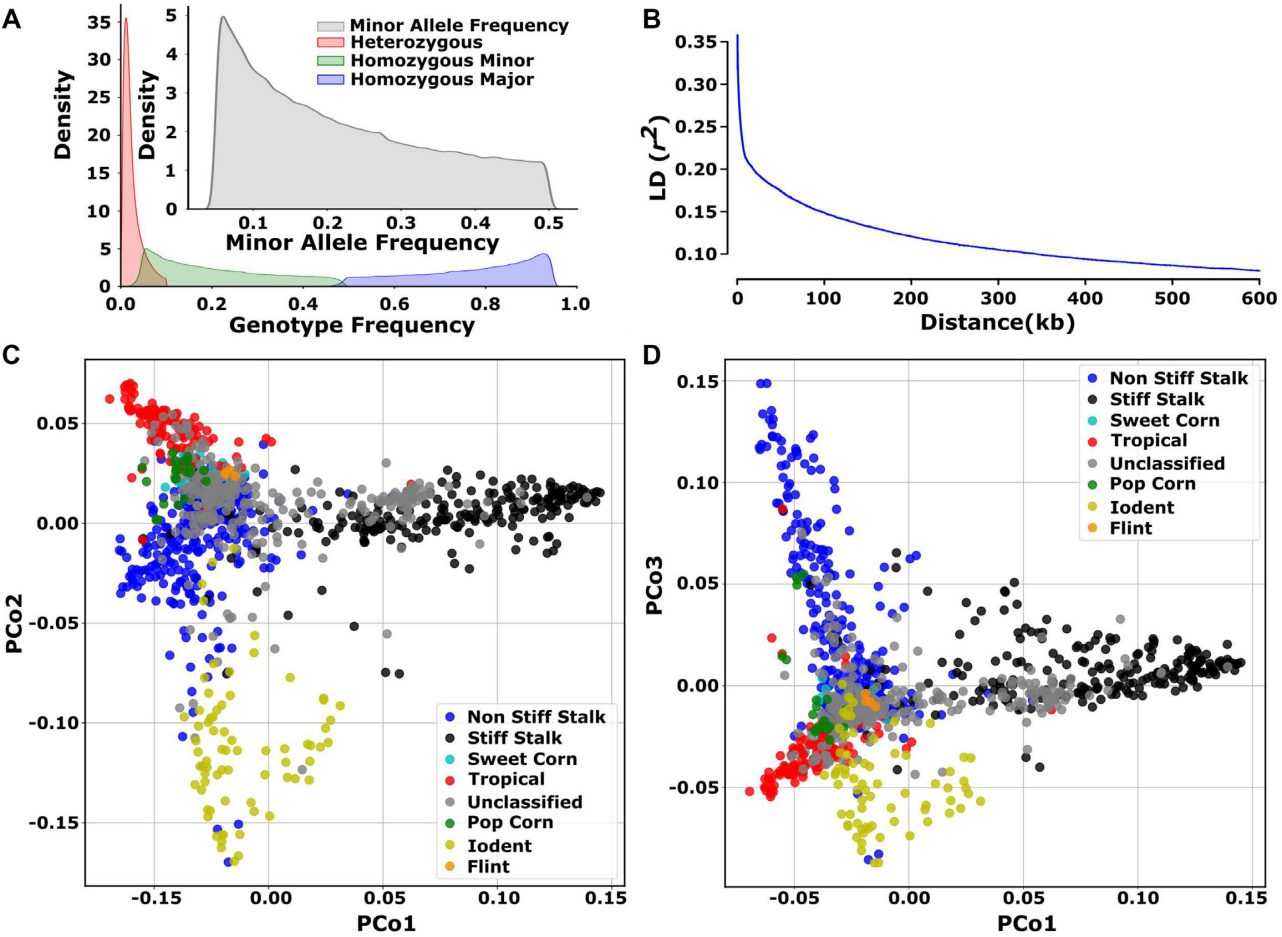
Characteristics of Maize Association Panel Marker data sets. (A) Genotype frequency and minor allele frequency of the marker data set. (B) The genome-wide LD decay with maximum distance of 600 kilobases between 2 SNPs. (C) Genetic relationship among the accessions used in this study and visualized using multidimensional scaling/principal coordinate analysis of the distance matrix. The x- and y-axes represent first and second principal component coordinates. Each point is color coded by the heterotic group each accession belongs to. (D) Genetic relationship among the accessions used in this study and visualized using multidimensional scaling/principal coordinate analysis of the distance matrix. The x- and y-axes represent first and third principal component coordinates. Each point is color coded by the heterotic group each accession belongs to.

The 17,717,568 polymorphic markers chosen for downstream analysis were distributed roughly evenly across the 10 chromosomes of maize, with local reductions in SNP density around centromeres/pericentromeric regions of each chromosome (Supplementary Fig. S3A). Rare SNPs with minor allele frequencies <0.1 were modestly more abundant than common SNPs (Fig. 2A). Linkage disequilibrium decayed rapidly, with the average *r*^2^ between 2 SNPs separated by 10 kilobases being approximately 0.18 (Fig. 2B), similar to previous reports [21, 22]. The first 3 principal components of variation explained approximately 10% of total variance among genotypes (Supplementary Fig. S3B). Principal coordinate (PCo) analysis using this SNP set separated lines with known assignments to major heterotic groups (Fig. 2C, D). The same set of PCo analyses did not identify obvious biases in the distribution of lines present in different association panels (Supplementary Fig. S3C).

### Unified marker-trait analyses

**Figure 3:**
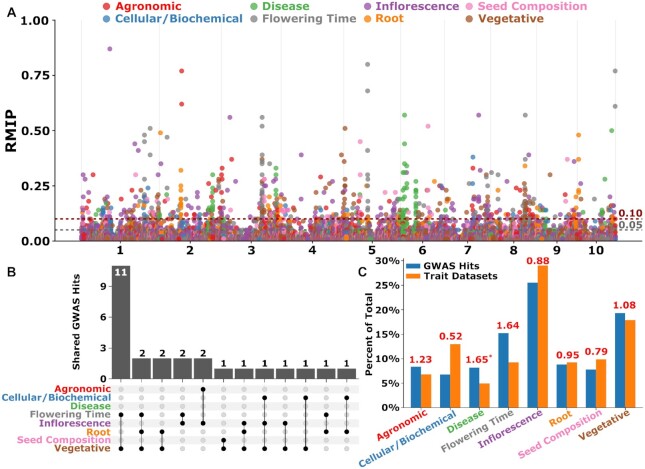
GWAS summary: multitrait peaks detected across phenotypic categories. (A) Combined Manhattan plot for GWAS using all 1,014 individuals screened using 18M markers. Dashed gray and red lines indicate the cutoff of 5% and 10% for statistical significance calculated based on RMIP value. Each chromosome is shown in the x-axis. The y-axis is the RMIP values ranging from 0 to 1. (B) An upset plot showing number of shared GWAS hits between various phenotypic categories. (C) Percent representation of GWAS hits for the number of trait data sets analyzed. Number on top of each pair of bars in each phenotypic category corresponds to the ratio of GWAS hits/number of trait data sets analyzed in each category. Note: The ratio was higher for the disease traits, but the traits in this category are essentially the same trait analyzed at different time points in a time-series manner; thus, most of the hits overlap among the traits, leading to an inflated ratio.

Genome-wide association studies conducted using FarmCPU with the 162 traits and about 18M markers described in the previous section and employing an RMIP cutoff of 5 for a suggestive association identified 2,154 signals across 151 traits (Fig. 3A). Among traits with 1 or more suggestively significant hits, the median number of hits was 12 (mean 12.57), the maximum was 33, and the minimum was 1.

Consolidation of 2,154 SNPs with at least suggestive statistically significant associations with phenotypes (≥5 RMIP) into distinct peaks based on physical distance and linkage disequilibrium (LD) (see Materials and Methods) reduced the number of associations to 1,466 peaks distributed across phenotypes assigned to 8 categories (Table 2). Of these 1,466 peaks, 161 peaks were associated with 11 agronomic traits, 92 peaks were associated with 17 of the 21 total cellular/biochemical traits, 72 peaks were associated with 8 disease traits, 176 peaks were associated with 15 flowering time traits, 459 peaks were associated with 41 of 47 total inflorescence traits, and 113 peaks were associated with 15 root traits, 128 with 16 seed composition traits, and 295 with 28 of 29 total vegetative traits (Fig. 3B and Table 2).

**Table 2:** Summary of unique associations with RMIP ≥5 within each of the 8 phenotypic groups analyzed

Phenotype group	No. of phenotypes analyzed	No. of phenotypes with hits	No. of peaks	No. of single trait peaks	No. of multitrait peaks	No. of multitrait peaks within each category^a^	No. of peaks associated across each category
Agronomic	11	11	161	155	6	4	2
Cellular/Biochemical	21	17	92	69	23	20	3
Disease	8	8	72	44	28	28	0
Flowering Time	15	15	176	128	48	32	16
Inflorescence	47	41	459	420	39	32	7
Root	15	15	113	81	32	25	7
Seed Composition	16	16	128	108	20	19	1
Vegetative	29	28	295	247	48	28	20
Total Unique	162	151	1,466^b^	1,252	214^b^	188	26^b^

^a^Excluding 26 peaks that overlap between 2 or more phenotype groups/categories. Of these 26 peaks, 22 are associated with traits belonging to 2 phenotype categories and 4 peaks are associated with phenotype traits belonging to 3 phenotype categories.

^b^The total unique value is less than the sum of all values in respective columns because some of the peaks were associated with phenotypes in multiple categories and they are depicted in each category they show significance.

**Figure 4:**
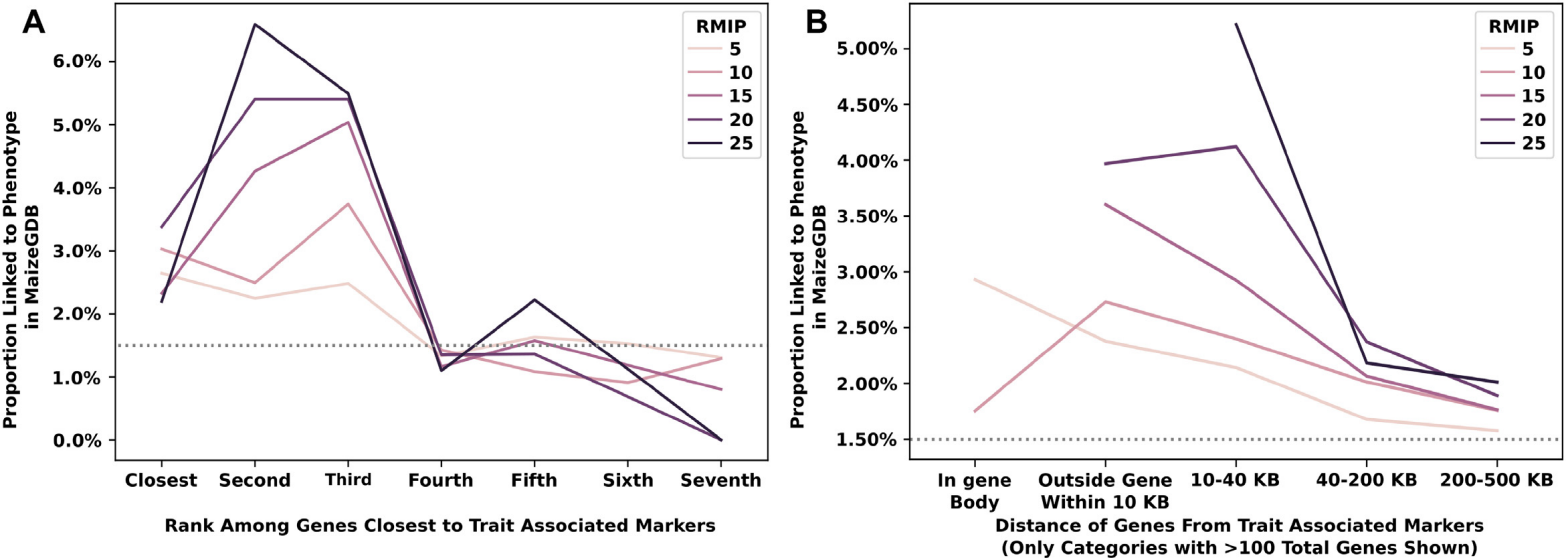
Probability of genes at different distances from peak SNP from GWAS is linked to phenotypes. (A) Gene positions of unique trait associations. First 7 genes closest to the GWAS peaks were selected and shown on the x-axis. (B) Gene order of unique trait associations. The distance of the genes from the trait-associated markers is shown on the x-axis.

A wide range of approaches are employed in the literature to define the set of annotated gene models adjacent to a significant GWAS peak, which should be labeled as “candidate genes.” These can include both fixed windows around the peak, examining an arbitrary number of the closest annotated gene models to the peak, or adaptive windows defined based on local levels of linkage disequilibrium or haplotype blocks. To assess the precision provided by the peaks identified in this study, we utilized a set of 604 gene models recorded in the MaizeGDB database [35] as associated with 1 or more phenotypes. These gene models constituted 1.5% of the total set of 39,498 annotated gene models present on the B73_v4 reference genome [20]. The first 3 genes closest to GWAS peaks identified above were more likely to be associated with reports of phenotypes in MaizeGDB than the expected background rate, and this pattern became stronger at more stringent RMIP cutoffs (Fig. 4A). When employing physical distance rather than rank order, the greatest enrichment of genes with reported phenotypes in the MaizeGDB database was observed in the categories “within gene,” “closer than 10 kilobases,” and “10–40 kilobases,” although noticeable enrichment remained observable at greater distances from the GWAS peak (Fig. 4B).

**Figure 5:**
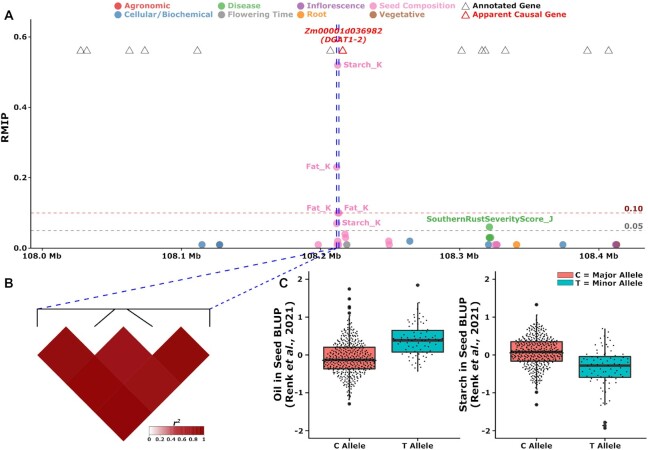
Combined GWAS identifies peak associated with seed starch and fat. (A) View of resampling marker inclusion probability values for markers in a window from 108,211,603 to 108,213,234 on chromosome 6 spanning 200 kilobases upstream and downstream of the pleiotropic peak identified for seed starch and oil content. Only markers with resampling marker inclusion probability values ≥0.01 are shown. (B) The LD relationships between the significant SNPs within the peak. (C) Distributions of observed oil and starch content values reported in [32] for lines carrying either allele of the peak SNP located at position 108,212,338 bp.

Flowering time trait data sets tended to identify a disproportionately high number of independent GWAS peaks (Fig. 3C), potentially as a result of the greater proportion of variance among these traits explained by genetic factors (Fig. 1E). Overall, in 1,252 cases (85.4%), a peak was identified only in the analysis of a single trait data set (Table 2). The remaining 214 peaks were identified in analyses of 2 or more separate trait data sets. In 188 cases, the same peak was identified in the analysis of 2 or more phenotypes belonging to the same general category. For example, a peak consisting of 4 SNPs in high LD with each other on chromosome 6 spanning from 108,211,603 to 108,213,234 bp, with the single highest RMIP SNP located at 108,212,338 bp, was identified in analysis of both kernel starch abundance (Starch_K) (RMIP = 52) and kernel fat abundance (Fat_K) (RMIP = 23) within the overall category of “seed composition” traits [32]. The peak spans the 5′ end of the gene model Zm00001d036982 (108,212,462 to 108,219,350 on chromosome 6), which encodes *DGAT1-2* (diacylglycerol O-acyltransferase 1-2)/*ln1* (linoleic acid1). *DGAT1-2* substantially increases the seed oil and oleic acid contents [36]. Largely, oils are stored in the form of triacylglycerol (TAG), and DGAT catalyzes the final step of TAG biosynthesis by transferring an acyl group from acyl-CoA to the sn-3 position of 1,2-diacylglycerol (DAG), thus acting as the rate-limiting enzymes for TAG biosynthesis [37,38] (Fig. 5A, B). The rarer allele (“T”) is associated with an increase in seed fat and decreases in seed starch (Fig. 5C). The starch-promoting allele was more abundant in iodent subpopulations and less abundant in sweet corn subpopulations (Supplementary Fig. S4). The original analysis of these 2 data sets employed the FarmCPU algorithm, but without resampling [32], and did not identify these 2 associations, consistent with observed RMIP values, which suggest only a 1 in 2 chance of detecting the DGAT/starch association and only a 1 in 4 chance of detecting the DGAT/fat association in a single round of GWAS.

In the remaining 26 cases where the same genomic interval was identified in the analysis of multiple trait data sets (Fig. 6A–D, Supplementary Figs. S5–S26), the trait data sets involved spanned 2 or more categories, with 22 peaks associated with trait data sets spanning 2 categories and 4 peaks associated with trait data sets spanning 3 categories (Fig. 3B). Genomic intervals associated with flowering time were disproportionately more likely to be associated with phenotypes from at least 1 other category. Sixteen of 176 unique peaks identified for flowering time were also associated with 1 or more phenotypes from other categories (9%), while only 10 of 1,290 unique peaks (0.8%) identified for nonflowering time traits were associated with traits from 2 or more of the remaining 7 categories (Fig. 3B).

**Figure 6:**
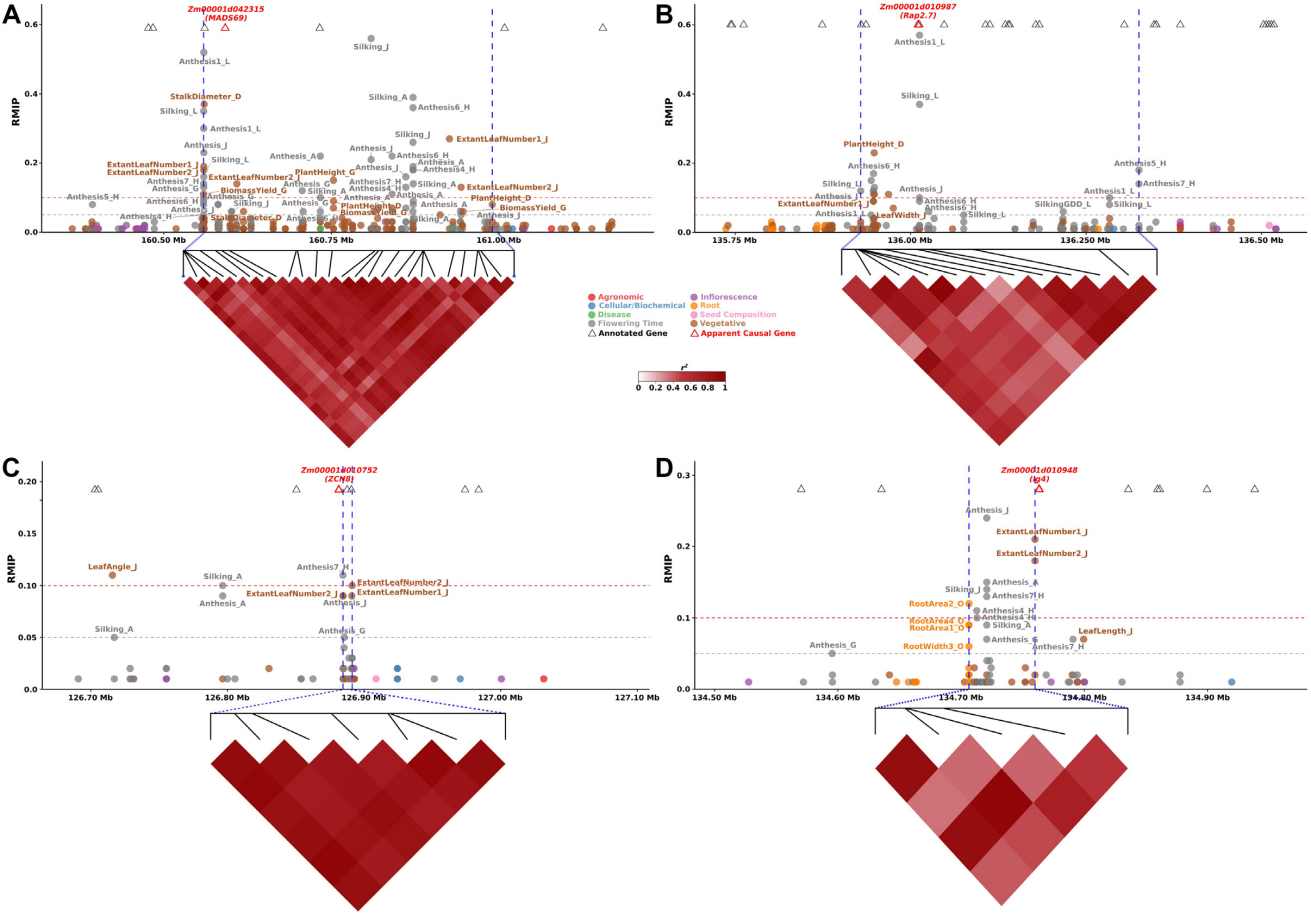
GWAS peaks associated with multiple traits. (A) Local Manhattan plot with ±200 kilobases of pleiotropic peak on chromosome 3 from 160,559,294 to 160,989,691 bp. This peak is associated with *MADS69* (Zm00001d042315). The phenotypes associated with this peak belongs to Flowering Time and Vegetative categories. The phenotypes associated with this peak are Anthesis1_L, Anthesis4_H, Anthesis6_H, Anthesis7_H, Anthesis_A, Anthesis_G, Anthesis_J, BiomassYield_G, ExtantLeafNumber1_J, ExtantLeafNumber2_J, PlantHeight_D, PlantHeight_G, Silking_A, Silking_J, Silking_L, and StalkDiameter_D. The vertical dashed lines show the peak boundary. (B) Local Manhattan plot with ±200 kilobases of pleiotropic peak on chromosome 8 from 135,928,821 to 136,325,345 bp. This peak is associated with *Rap2.7* (Zm00001d010987). The phenotypes associated with this peak belong to Flowering Time and Vegetative categories. The phenotypes associated with this peak are Anthesis1_L, Anthesis5_H, Anthesis6_H, Anthesis7_H, Anthesis_A, Anthesis_G, Anthesis_J, ExtantLeafNumber1_J, LeafWidth_J, PlantHeight_D, SilkingGDD_L, and Silking_L. The vertical dashed lines show the peak boundary. (C) Local Manhattan plot with ±200 kilobases of pleiotropic peak on chromosome 8 from 126,884,534 to 126,891,234 bp. This peak is associated with *ZCN8* (Zm00001d010752). The phenotypes associated with this peak belong to Flowering Time and Vegetative categories. The phenotypes associated with this peak are Anthesis7_H, Anthesis_G, Anthesis_J, ExtantLeafNumber1_J, and ExtantLeafNumber2_J. The vertical dashed lines show the peak boundary. (D) Local Manhattan plot with ±200 kilobases of pleiotropic peak on chromosome 8 from 134,706,389 to 134,759,977 bp. This peak is associated with *lg4* (Zm00001d010948). The phenotypes associated with this peak belong to Flowering Time, Root, and Vegetative categories. The phenotypes associated with this peak are Anthesis4_H, Anthesis7_H, Anthesis_A, Anthesis_G, Anthesis_J, ExtantLeafNumber1_J, ExtantLeafNumber2_J, RootArea1_O, RootArea2_O, RootArea4_O, RootWidth3_O, Silking_A, and Silking_J. The vertical dashed lines show the peak boundary.

An illustrative example of the potential for genes influencing flowering time to be identified in genome-wide association studies for other traits is the case of *ZmMADS69. ZmMADS69* (syn *Zmm22*) (Zm00001d042315) is a MADS-box transcription factor located between 160,564,021 bp and 160,591,933 bp on maize chromosome 3, which has been shown to function as a flowering activator, with a derived allele conferring earlier flowering in many maize lines relative to its wild progenitor teosinte [8, 39]. A peak consisting of 26 SNPs in high LD with each other was consistently identified for multiple flowering time–related traits, including 7 measurements of anthesis (male flowering) in different environments (Anthesis_A, Anthesis_G, Anthesis1_L, Anthesis4_H, Anthesis6_H, Anthesis7_H, Anthesis_J) and 3 measures of silking in different environments (Silking_A, Silking_L, Silking_J). The same peak was also identified in the analysis of multiple vegetative traits, including measurements of plant height in 2 environments (PlantHeight_D, PlantHeight_G), extant leaf number, stalk diameter, and biomass yield (Fig. 6A). *ZmMADS69* has been shown to downregulate the expression of *ZmRap2.7*, which relieves repression of the florigen gene *ZCN8*, causing/resulting in early flowering [39]. Both *ZmRap2.7* and *ZCN8* are located on chromosome 8 [40–42], and both of these genes are also associated with GWAS peaks. A peak on chromosome 8 consisting of 4 SNPs between 126,884,534 bp and 126,891,234 bp was separated by only 2 kilobases from the gene model encoding *ZCN8* (Zm00001d010752, located between 126,880,531 and 126,882,389 bp) and was associated with anthesis in 3 environments (Anthesis_G, Anthesis7_H, Anthesis_J). *ZmRap2.7* (Zm00001d010987) is located approximately 10 megabases away from *ZCN8* on chromosome 8 (between 136,009,216 and 136,012,084 bp) and is associated with a peak consisting of 13 SNPs that was detected for 7 measurements of anthesis (Anthesis_A, Anthesis_G, Anthesis1_L, Anthesis5_H, Anthesis6_H, Anthesis7_H, Anthesis_J), 2 approaches to measuring silking in the same environment (Silking_L, SilkingGDD_L), and a number of vegetative traits, including extant leaf number (ExtantLeafNumber1_J), leaf width (LeafWidth_J), and plant height (PlantHeight_D).

In addition to the 3 peaks discussed above, 13 other peaks were also associated with both flowering time traits and traits from other categories (Fig. 3A, B and Supplementary Table S4). In 2 cases, a significant signal for flowering time was colocated with significant signals for inflorescence architecture traits. The first, located on chromosome 1 between 102,077,749 and 102,120,437 bp, consists of 2 SNPs in high LD with each other and is significantly associated with both date of silking (Silking_J) and ear length (EarLength_O) in separate environments (Supplementary Table S4 and Supplementary Fig. S5). The second, located on chromosome 4 between 78,020,118 and 78,451,569 bp, consists of 2 SNPs and showed significant associations with both male flowering in one environment (Anthesis5_H) and the length of the central spike of the tassel in another environment (SpikeLength1_C) (Supplementary Table S4 and Supplementary Fig. S14). In 11 cases, a significant association for flowering time was colocated in the genome with a signal for an above-ground vegetative trait data set. These were typically vegetative traits with known links to flowering time, including leaf/node number and plant or ear height (Supplementary Table S4, Fig. 6D, and Supplementary Figs. S6, S12, S16–S20, S22, S24, and S26).

The potential pleiotropy of genes linked to flowering time was not confined to above-ground traits. In 3 cases, a significant signal for flowering time was also associated with 1 or more data sets describing variation in root phenotypes. A signal on chromosome 5 between 94,710,702 and 94,712,951 bp was associated with flowering time across a wide range of environments (Anthesis_A, Anthesis1_L, Anthesis7_H, Anthesis_J, Silking_J, Silking_L, SilkingGDD_L), with other above-ground vegetative traits (LeafAreaIndex_J, LeafLength_J) and with many root architecture traits (RootArea1_O, RootArea2_O, RootArea4_O, RootWidth4_O) (Supplementary Table S4 and Supplementary Fig. S19). The specific SNPs that define the peak are all located within Zm00001d015513, which encodes a cinnamoyl-CoA reductase expressed primarily in leaves and leaf meristems [43]. A signal on chromosome 8 between 28,727,658 and 28,769,198 bp was associated with both male and female flowering time in Nebraska (Anthesis_J, Silking_J) and variation in root depth in Iowa (RootDepth1_O, RootDepth2_O) (Supplementary Table S4 and Supplementary Fig. S22). The last of the 3 signals associated with flowering time (Anthesis_A, Anthesis_G, Anthesis4_H, Anthesis7_H, Anthesis_J, Silking_A, Silking_J), leaf number, and root (RootArea1_O, RootArea2_O, RootArea4_O, RootWidth3_O) traits is also located on chromosome 8, between 134,706,389 and 134,759,977 bp. This 54-kilobase interval is entirely free of annotated genes but ends 600 bp upstream of classical mutant *liguleless4* (Zm00001d010948) (Fig. 6D). *Liguleless4* (synonym *knox11*) encodes a knox transcription factor that is highly expressed in the SAM, seed radicle, internode tissues, crown roots, pericarp of seed, and the endosperm of maize [43]. A dominant allele of *liguleless4* abolishes the ligule and alters the sheath-blade boundary in maize leaves [44] likely via ectopic expression [45], but phenotype of loss of function alleles, if any, remains uncharacterized.

## Discussion

The widespread adoption of diverse association panels in plant biology has enabled a wide range of research and discovery by researchers working on diverse phenotypes, species, and research questions [46]. Beyond lists of specific candidate genes identified in the main text or supplemental figures, the reuse of GWAS results can sometimes be challenging. Changes in genome versions, gene model annotations, or genetic marker data sets, as well as changes in best practices and algorithms for conducting genome-wide association tests, can all hinder comparisons with and/or reuse of previously published GWAS results. In the field of human genetics where the release of individual-level trait and genetic data could raise privacy and ethics concerns, the field has converged on a standard of releasing summary statistics but not individual-level trait and genetic data [47]. Working with plant data, privacy concerns do not typically preclude the release of individual-level data, and the reuse of the same genotypes across independent studies increases the potential value of individual-level data.

We identified 21 published papers describing phenotypes or GWAS conducted using 1 or more of 2 widely adopted association panels in maize [3,4]. In 18 cases (85%) it was possible to locate raw trait values for individual lines used in the published analyses, including the 16 studies summarized in Table 1 and the 2 additional papers not included in our analyses. The high frequency with which trait data are being released for published GWAS studies is encouraging as it indicates the maize quantitative genetics community is adopting similar norms and practices to the genomics community, which has long been a leader in promoting strong best practices for raw data deposition and dissemination [48]. Unlike the genomics community, the plant quantitative genetics community does not have access to widely used and standardized data repositories. Challenges in integration of these data included inconsistent naming, extra lines that were not part of the panel, repeated traits across papers, and data distributed across supplemental files or Figshare. Metadata for how and when individual traits were collected typically were present but often needed to be manually extracted from reading the manuscript text. Information that would further increase the value of released trait data such as the GPS coordinates and planting dates of individual field trials was provided in some cases but not others. The identification of a single common repository, standards for metadata on individual field trials, and for the preservation of a single unique identifier for each genotype included in a community association population would all lower barriers to the reuse of trait data sets. However, despite these current challenges, both the overall consistency and quality of data release and documentation was exceptional, enabling the investigation of multienvironment and multitrait genetic associations.

One desirable outcome of having access to raw trait data is that it enables reanalysis of existing trait data sets, each of which represents a substantial investment of both finance resources and human effort/suffering, as new higher-resolution genetic marker data sets and new analysis algorithms become available. Here we employed a RMIP-based filter to the FarmCPU GWAS algorithm [11,14, 49] and were able to identify 2,154 suggestive associations (RMIP ≥5) and 697 confident associations (RMIP ≥10) across 162 traits collected in 33 environments spanning at least 7 states. These signals included new associations identified as a result of either new genetic marker data and/or the FarmCPU/RMIP approach (Fig. 5). Overall, 1,466 and 468 unique sites in the genome were tagged with a suggestive or confident association, respectively. These associations were enriched near genes with previously reported phenotypic effects (Fig. 4). However, many of these signals are located near genes whose functions were previously entirely unknown or estimated purely via functional data on homologs, which is typically useful for inferring molecular function of a protein encoded by a given gene but can produce misleading information on the specific biological processes a given gene is involved in [50]. Many traits, particularly flowering time, were collected repeatedly in different studies conducted in different environments. While some signals were consistently identified for the same trait across multiple independently collections, others were statistically significant in only a single environment. This second class of association likely includes loci with significant genotype-by-environment interactions, loci with modestly smaller effect sizes and/or minor allele frequencies that will sometimes fall above the threshold for statistical significance and sometimes below, and false-positive associations. Ultimately, distinguishing between these categories, as well as further characterizing patterns of genotype-by-environment interaction, would further increase the value of this data set. However, simply including data from multiple environments and experiments already enables the community to evaluate which trait-associated loci produce consistent and stable results and are likely to be more amenable to detailed genetic characterization. These new layers of functional data will be most useful if they can be integrated into community genomics repositories. In this case, the functional data generated as part of this project have been integrated as browser tracks and downloads at the maize community repository MaizeGDB [35] to enable maize researchers to quickly access these data, cross-reference them with other data types, and compare with mutant or QTL mapping results.

A straightforward way to increase power and utility of reanalyzing data from community association panels is through the integration of data from additional widely employed community association panels. The AM508 panel discussed above [5,51] has been employed in studies of seed composition [51–54], morphological and yield-related traits [55], biomass accumulations [56], and both biotic [57,58] and abiotic stress tolerance [59]. Many studies of this population employed a set of markers generated through a combination of microarray and RNA-seq–based genotyping and anchored to the B73_RefGen_V2 reference genome [51]. However, several years ago, this panel was resequenced to an average depth of 20× [60], creating the potential to generate unified genetic marker data sets incorporating both the 500 maize inbreds of the AM508 panel and the 1,000 maize inbreds present in the union of the SAM and WiDiv panels. One analytical challenge extension to the AM508 association panel or other maize association panels is the lack of the large intersection set of shared genotypes present between the SAM and WiDiv panels as a result of the role the MAP panel played in the origin of both of these populations.

The significant signals identified for flowering time and both above-ground and below-ground plant architectural traits adjacent to *liguleless4*/*knox11* are an example of an intermediate case between confirmation of known functions and assigning potential functions to previously uncharacterized genes or regions of the genome. *Liguleless4* belongs to the knox class I gene family [45], a family of genes involved in regulating plant development via expression in apical meristems [61]. The *liguleless4* gene itself was identified via a dominant allele *Lg4-O*, which alters development at the leaf blade/sheath boundary [44] and is associated with ectopic expression [45]. Loss-of-function alleles of the *liguleless3* gene, a paralog of *liguleless4*, do not exhibit any obvious phenotype [45]. A role for *liguleless4* in determining flowering time and above/below-ground plant architecture is consistent with the reported expression pattern of the wild-type allele in tissues, including the shoot apex, root tips, and developing inflorescence [45]. The MADS69 gene was previously speculated to be pleiotropic for multiple traits [39] and indeed exhibited pleiotropic associations with variation in multiple traits in our study. Similarly, the well-characterized genes ZCN8 and Rap2.7, which also play a role in determining flowering time in maize, exhibited pleiotropic associations in this study. This study identified a number of genes associated with both above- and below-ground traits. These include *liguleless4*, Zm00001d015513 (Chr5:94710702..94712951), which was associated with variation in anthesis, silking, leaf area index, leaf length, and various traits related to root architecture. The other potential gene for future characterization is Zm00001d008987 (Chr8:28727658..28769198), a previously uncharacterized putative costunolide 3-hydroxylase associated with both flowering time and root depth traits.

One striking observation from the colocalization of association signals across multiple trait data sets was how common the reidentification of shared signals was. In total, 14.5% (214/1,466) of all suggestive associations and 16.2% (76/468) of all confident associations were identified in at least 2 trait data sets. One utility of reanalyzing published trait data sets is that variants with consistent but moderate effects across many studies can be distinguished from, and assigned higher confidence, than signals of equivalent statistical significance, which are identified in only a single study in a single environment. Another lesson to take away from the same colocalization data is how common it was for the same locus to be identified for traits belonging to separate categories of phenotypes. The interpretation of a genetic locus with a significant association with root area will be quite different depending on whether that same locus is also associated with flowering time [62] (Fig. 6D and Supplementary Figs. S19 and S22). In both cases, the key takeaway is that researchers do not have to analyze or interpret GWAS in a vacuum but instead are able to interpret their results in the context of the rich data sets of previously scored phenotypes and previous GWAS analyses. Our understandings of genetics, genotype-by-environment interactions, and pleiotropy will all benefit from the broad use of these rich data sets.

## Potential implications

Logistical and financial limitations often constrain quantitative genetic analyses of plant populations to collecting data on a single phenotype or a small suite of related phenotypes, limiting our capacity to identify and study the ways individual genetic variants can control multiple phenotypic outcomes. The data set described in this article, including 162 traits scored across different subsets of 1,014 immortalized maize inbred genotypes and associated with a high-density marker set of 18M segregating markers, dramatically lowers the barriers for further quantitative genetic studies of both pleiotropy and genotype-by-environment interactions in maize. In addition, by identifying more than 2,000 confident or suggestive genetic associations in the maize genome, this data set means researchers do not have to analyze or interpret GWAS in a vacuum but instead can interpret their results in the context of the rich data sets of previously scored phenotypes and previous GWAS analyses.

## Materials and Methods

### Collection of published trait data

Papers publishing maize analyses were identified by searching papers citing the initial description of the first iteration of the WiDiv panel [4], the initial description of the SAM diversity panel [3], or the expanded WiDiv panel [8]. Screening of studies citing 1 or more of these papers concluded on 25 June 2021. Published studies were excluded if we were unable to locate de-anonymized trait values for individual accessions or if fewer than 200 total accessions were phenotyped. If 2 studies indicated that the same trait was collected from the same lines in the same location in the same year, only 1 version of the data was retained. If a study published both data from individual environments and aggregated estimates across environments (e.g., averages, best linear unbiased predictions [BLUPs], or best linear unbiased estimates), only individual environment trait data were retained. If only aggregate estimates across environments were published, aggregated traits were employed. After preliminary analysis with MLM-based GWAS, several other trait data sets were discarded when it proved impossible to effectively control false discoveries across the genome. The final data file of all accession-level trait values employed in this study is provided as Supplementary Table S2.

### Trait data not previously published

A set of 752 maize genotypes, which were a strict subset of the WiDiv panel and included 254 of 369 genotypes from the SAM diversity panel, was evaluated in a field experiment conducted in Lincoln, Nebraska, in the summer of 2020. The experimental design of this field experiment has been previously described [13]. Briefly, the field was laid out in a randomized complete block design with 2 blocks of 840 plots, including a repeated check genotype (B97). Each plot was 2 rows, 7.5 feet (approximately 2.3 meters) long with 30-inch row spacing (approximately 0.76 meters), 4.5-inch spacing between sequential plants (approximately 11.5 centimeters), and 30-inch alleyways between sequential plots (approximately 0.76 meters). The field was planted on 6 May 2020 and was located at the University of Nebraska–Lincoln’s Havelock Farm (40.852 N, 96.616 W).

Tassel architecture phenotypes were collected once tassels had fully emerged for 3 randomly selected plants per plot, avoiding edge plants. Tassel lengths were measured from the basal primary tassel branch to the tip of the tassel spike. Branch zone length was defined as the length from the basal primary tassel branch to the top primary tassel branch. Tassel spike length was defined as the length from the top primary tassel branch to the tip of the tassel spike. The total number of primary tassel branches was also counted as well as the number of these primary tassel branches that were initiated but later aborted (Supplementary Fig. S1).

Male and female flowering times for each plot were scored on the first day that 50% of plants had visible pollen shed or visible silks, respectively. Root and stalk lodging were scored at the end of the growing season as a percentage of extant plants in the plot, following the published Genomes to Fields phenotyping protocol for both traits [63]. Leaf phenotypes—leaf length, leaf width, and leaf angle—were measured for 2 plants per plot and collected from each plot after anthesis and silking. One plant was randomly selected from each of the 2 rows for measurement, avoiding edge plants when possible. Leaf length was measured from the leaf ligule to the leaf tip on the adaxial surface of the first leaf above the top ear of the plant. Leaf width was measured on the same leaf at the midpoint between the ligule and the leaf tip. Extant leaf number was determined by counting the number of visible leaf collars on the same 2 plants. Plant height was measured between the soil surface and the flag leaf collar using a marked pole. Leaf Area Index values were estimated using a LAI-2200C Plant Canopy analyzer (LI-COR, Inc. Lincoln, Nebraska, USA). For each plot, 1 above-canopy and 3 below-canopy measurements were collected using the LAI-2200C’s 270-degree view cap. The 3 below-canopy measurements were collected diagonally in the space between the 2 rows of the plot. The first measure was adjacent to 1 row, the second equidistant between the rows, and the third adjacent to the second row. Leaf Area Index measurements were collected between 28 July and 12 August 2020.

All ears were harvested from 8 semi-randomly selected plants per plot with edge plants being excluded when possible. Ear length, ear width, length of fill, kernel row number, and number of kernels per row were hand measured or hand counted for 6 ears per plot or all ears when fewer than 6 ears were present (Supplementary Fig. S2). The average of individual ears was used to calculate plot-level values. All harvested ears were weighed and shelled, and the resulting pooled grain was also weighed. Cob weight was calculated as the difference between ear weight and grain weight. Initial hundred kernel weight was calculated by counting and weighing 100 kernels per plot after shelling and pooling of grain. Grain moisture was measured using a Dickey-John GAC® 2500-AGRI Grain Analysis Computer (Dickey-John® Corporation, Auburn, IL, USA). Total grain weight and hundred kernel weight were recalculated to a standardized 15.5% moisture content. When insufficient grain was harvested to collect accurate grain moisture data using the GAC® 2500-AGRI, a default value of 8.25% moisture, corresponding to the approximate median of all grain moisture values, was employed to calculate moisture-standardized grain weight and hundred kernel weight. Further, the BLUPs for each phenotype were calculated by fitting a linear mixed model using R package lme4 [64] with genotypes fit as the random variable for the traits with data from 2020.

### Unified genetic marker data

A single set of markers scored across 1,049 accessions was employed for downstream analyses. These genotypes were determined based on marker data aggregated from 3 published sources: resequencing data for the WiDiv-503 panel (454 individuals) [18], resequencing data generated as part of the HapMap3 project (141 individuals) [9], and RNA-seq data for the WiDiv-942 panel (399 individuals) [7, 8]. The specific NCBI SRA ID numbers of the files used to call SNPs for each of the accessions are provided in Supplementary Table S2.

Both genome resequencing data and RNA-seq data were quality trimmed using Trimmomatic (v0.33) [65]. BWA-MEM (v0.7) with default parameter settings [66] was employed to align the resulting trimmed resequencing data to v4 of the B73 maize reference genome [19, 20]. STAR (v2.7) [67] was used to align the trimmed RNA-seq reads to v4 of the B73 maize reference genome in 2 rounds as described in Sun et al. [68]. Apparent polymerase chain reaction duplicates were marked within the resulting BAM alignments using picard (v2.22) [69]. *A priori* segregating genetic markers identified in maize HapMap3 [9, 13] were scored for each individual using the GATK toolkit (v5.1) [70]. Missing values were imputed using beagle/5.01 with the HapMap3 population treated as a reference panel and the following parameter settings: window = 1, overlap = 0.1, ne = 1200 [71]. The imputed genetic marker data set was filtered to remove markers with a minor allele frequency less than 0.01 or proportion of site heterozygous calls greater than 0.1 to produce the final set of 17,717,568 SNP markers.

### Quantitative genetic analysis of trait data

A kinship matrix for the complete set of 1,049 genotyped accessions, including all maize lines included in the SAM or WiDiv panels and 35 additional maize lines for which sequence data were generated and released as part of Mazaheri et al. [8], was calculated using the first method described by VanRaden [72] as implemented in rMVP (v1.0.5) [73]. Narrow-sense heritability for each trait was calculated using this kinship matrix and the R package sommer (v4.1.1) [74]. Multidimensional scaling or the PCo analysis was performed with –mds-plot 2 and –cluster options within plink v1.90 [75]. Genome-wide patterns of linkage disequilibrium decay were estimated by calculating (LD/*r*^2^) for all pairs of genetic markers where both genetic markers exhibited minor allele frequency greater than 0.05 and were separated up to a physical distance of less than 600 kilobases using PopLDdecay (v3.41) [76].

Marker-trait associations were identified using 100 iterations of the FarmCPU algorithm as implemented in the R package rMVP v1.0.5 with parameter settings maxLoop = 10; method.bin = “FaST-LMM” [11, 73]. For each iteration, the first 3 principal components calculated from the genetic marker data set were used as a fixed effect, and the kinship matrix calculated internally by the FarmCPU algorithm was fitted as a random effect. As different trait data sets contained missing data for different individuals, analyses for each trait data set were conducted by subsetting the marker file separately for each trait analyzed to generate a new file containing only marker data for the individuals with nonmissing phenotypic values, and analyses of different traits included data from a different number of individuals with nonmissing phenotypic values. The overall marker file was filtered on a per-GWAS basis to retain only those markers with a minor allele frequency >0.05 among the lines phenotyped for a given trait prior to association testing for that trait. For each trait, 100 analyses were run, each incorporating data from a different randomly selected subset of phenotyped lines [14]. In each resampling analysis, the overall threshold for statistical significance was the Bonferroni corrected *P*value at 5%. RMIP values for each marker were calculated as the proportion of the 100 analyses in which that marker was significantly associated with the target trait [14, 49, 77–79].

Linkage disequilibrium was calculated among all genetic markers with RMIP values ≥5 for at least 1 trait. Genetic markers with linkage disequilibrium >0.5 were merged into single peaks for downstream analyses, unless the markers were separated by >1 Mb. When 2 or more markers were merged into a single peak, the marker with the greatest RMIP value was selected as representative of the entire peak.

## Availability of Supporting Data and Materials

Phenotypic values for all trait data sets employed in this study for all maize accessions evaluated are provided in Supplementary Tables S2 and S3. The sources of sequence data used to call genetic marker genotypes for each maize accession were NCBI BioProjects PRJNA661271, PRJNA189400, and PRJNA437324 [7, 8, 10]. The specific SRA IDs for individual maize accessions are indicated in Supplementary Table S2. The locations of GWAS peaks, the traits associated with each peak, and the genes adjacent to each peak are provided in Supplementary Tables S4 and S5. The VCF file used for genetic analyses has been deposited at FigShare [80]. Scripts and code used to implement various analyses described in the methods section above have been deposited in GitHub [81]. All supporting data and materials are available in the GigaScience GigaDB database [82].

### Additional Files

**Supplementary Fig. S1**. Phenotyping of tassel architecture. (A) Tassel lengths were measured from the bottom-most primary tassel branch to the tip of the tassel spike (red bars). (B) The branch zone length was defined as the length from the bottom-most primary tassel branch to the upper-most primary tassel branch (red left-facing bracket). (C) The tassel spike length was defined as the length from the upper-most primary tassel branch to the tip of the tassel spike (red right-facing bracket). (D) The total number of primary tassel branches was also counted (example arrowed) as well as the number of these primary tassel branches that were initiated but later aborted (not pictured).

**Supplementary Fig. S2**. Phenotyping of cob traits. (A) Ear lengths were measured as distance from base to the tip of each cob in centimeters, highlighted in green. (B) Ear fill was measured as distance on the cob from base to the tip where the seeds were set, highlighted in red. (C) Ear width was measured as distance of diagonal of the cob, highlighted by blue. (D) Kernels per row correspond to the number of kernels on each line when cobs were set vertically, highlighted in pink. (E) Kernel row number corresponds to the number of rows, highlighted in brown.

**Supplementary Fig. S3**. Multidimension scaling or principal coordinate analysis. (A) Distribution of SNP density across the sorghum genome in 1-megabase sliding windows. (B) Scree plot of eigenvalues for the principal components estimated from the marker data used in this study. (C) Genetic relationship among the accessions used in this study and visualized using multidimensional scaling/principal coordinate analysis of the distance matrix. The x- and y-axes represent first and second principal component coordinates. Each point is color coded by the community association panels each accession belongs to.

**Supplementary Fig. S4**. Allele frequencies of the top SNPs associated with the DGAT-2 gene. (A) The allele frequency for the starch content in each subpopulation of top SNPs associated with the DGAT gene. (B) The allele frequency for the fat content in each subpopulation of top SNPs associated with the DGAT gene. (C) Percentage of alleles in each subpopulation: the starch-promoting allele was more abundant in iodent subpopulations and less abundant in sweet corn subpopulations.

**Supplementary Fig. S5**. Local Manhattan plot with ±200 kilobases of pleiotropic peak on chromosome 1 from 102,077,749 to 102,120,437 bp. This peak is associated with the phenotypes belonging to Inflorescence and Flowering Time categories. The phenotypes associated with this group are EarLength_O and Silking_J.

**Supplementary Fig. S6**. Local Manhattan plot with ±200 kilobases of pleiotropic peak on chromosome 1 from 276,657,865 to 277,120,054 bp. This peak is associated with the phenotypes belonging to Flowering Time and Vegetative categories. The phenotypes associated with this group are Anthesis1_L, Anthesis_J, ExtantLeafNumber1_J, ExtantLeafNumber2_J, Nodes_M, and Silking_J.

**Supplementary Fig. S7**. Local Manhattan plot with ±200 kilobases of pleiotropic peak on chromosome 1 from 280,994,269 to 281,039,919 bp. This peak is associated with the phenotypes belonging to Root and Vegetative categories. The phenotypes associated with this group are EarHeight_M and RootAngle2_O.

**Supplementary Fig. S8**. Local Manhattan plot with ±200 kilobases of pleiotropic peak on chromosome 2 from 82,811,901 to 82,811,901 bp. This peak is associated with the phenotypes belonging to Agronomic and Inflorescence categories. The phenotypes associated with this group are BushelAcreEquivalent_J and KernelsPerRow_J.

**Supplementary Fig. S9**. Local Manhattan plot with ±200 kilobases of pleiotropic peak on chromosome 2 from 88,191,911 to 88,201,317 bp. This peak is associated with the phenotypes belonging to Agronomic and Inflorescence categories. The phenotypes associated with this group are BushelAcreEquivalent_J, EarFilledLength_J, KernelsPerRow_J, and TotalGrainMassGrams_J.

**Supplementary Fig. S10**. Local Manhattan plot with ±200 kilobases of pleiotropic peak on chromosome 3 from 157,317,263 to 157,318,194 bp. This peak is associated with the phenotypes belonging to Root and Vegetative categories. The phenotypes associated with this group are EarHeight_M, RootAngle2_O, and RootWidth1_O.

**Supplementary Fig. S11**. Local Manhattan plot with ±200 kilobases of pleiotropic peak on chromosome 3 from 168,948,681 to 168,998,142 bp. This peak is associated with the phenotypes belonging to Cellular/Biochemical, Inflorescence, and Vegetative categories. The phenotypes associated with this group are BranchZoneLength_C, LeafCuticularConductance6_H, SpikeProportion_C, StalkDiamThin_N, and peri_N.

**Supplementary Fig. S12**. Local Manhattan plot with ±200 kilobases of pleiotropic peak on chromosome 3 from 218,796,811 to 218,832,925 bp. This peak is associated with the phenotypes belonging to Flowering Time and Vegetative categories. The phenotypes associated with this group are ExtantLeafNumber1_J and Silking_J.

**Supplementary Fig. S13**. Local Manhattan plot with ±200 kilobases of pleiotropic peak on chromosome 4 from 28,148,396 to 28,163,192 bp. This peak is associated with the phenotypes belonging to Inflorescence and Vegetative categories. The phenotypes associated with this group are Nodes_M and SkeletonLength_C.

**Supplementary Fig. S14**. Local Manhattan plot with ±200 kilobases of pleiotropic peak on chromosome 4 from 78,020,118 to 78,451,569 bp. This peak is associated with the phenotypes belonging to Flowering Time and Inflorescence categories. The phenotypes associated with this group are Anthesis5_H and SpikeLength1_C.

**Supplementary Fig. S15**. Local Manhattan plot with ±200 kilobases of pleiotropic peak on chromosome 4 from 190,332,784 to 190,410,017 bp. This peak is associated with the phenotypes belonging to Seed Composition and Vegetative categories. The phenotypes associated with this group are CrudeAsh_K, EarHeight_L, Ncombustion_K, Nkjeltec_K, PlantHeight_L, and Protein_K.

**Supplementary Fig. S16**. Local Manhattan plot with ±200 kilobases of pleiotropic peak on chromosome 5 from 4,615,668 to 4,617,726 bp. This peak is associated with the phenotypes belonging to Flowering Time and Vegetative categories. The phenotypes associated with this group are Anthesis1_L, EarHeight_L, and PlantHeight_L.

**Supplementary Fig. S17**. Local Manhattan plot with ±200 kilobases of pleiotropic peak on chromosome 5 from 32,944,052 to 32,957,580 bp. This peak is associated with the phenotypes belonging to Flowering Time and Vegetative categories. The phenotypes associated with this group are Anthesis4_H, Anthesis7_H, and ExtantLeafNumber2_J.

**Supplementary Fig. S18**. Local Manhattan plot with ±200 kilobases of pleiotropic peak on chromosome 5 from 39,053,843 to 39,077,552 bp. This peak is associated with the phenotypes belonging to Flowering Time and Vegetative categories. The phenotypes associated with this group are Anthesis_J and PlantHeight_G.

**Supplementary Fig. S19**. Local Manhattan plot with ±200 kilobases of pleiotropic peak on chromosome 5 from 94,710,702 to 94,712,951 bp. This peak is associated with the phenotypes belonging to Flowering Time, Root, and Vegetative categories. The phenotypes associated with this group are Anthesis1_L, Anthesis7_H, Anthesis_A, Anthesis_J, LeafAreaIndex_J, LeafLength_J, RootArea1_O, RootArea2_O, RootArea4_O, RootWidth4_O, SilkingGDD_L, Silking_J, and Silking_L.

**Supplementary Fig. S20**. Local Manhattan plot with ±200 kilobases of pleiotropic peak on chromosome 7 from 164,238,577 to 164,238,577 bp. This peak is associated with the phenotypes belonging to Flowering Time and Vegetative categories. The phenotypes associated with this group are Anthesis5_H and PlantHeight_J.

**Supplementary Fig. S21**. Local Manhattan plot with ±200 kilobases of pleiotropic peak on chromosome 7 from 167,280,764 to 167,280,878 bp. This peak is associated with the phenotypes belonging to Cellular/Biochemical and Root categories. The phenotypes associated with this group are LeafCuticularConductance6_H and RootAngle2_O.

**Supplementary Fig. S22**. Local Manhattan plot with ±200 kilobases of pleiotropic peak on chromosome 8 from 28,727,658 to 28,769,198 bp. This peak is associated with the phenotypes belonging to Flowering Time and Root categories. The phenotypes associated with this group are Anthesis_J, RootDepth1_O, RootDepth2_O, and Silking_J.

**Supplementary Fig. S23**. Local Manhattan plot with ±200 kilobases of pleiotropic peak on chromosome 8 from 86,775,550 to 87,443,426 bp. This peak is associated with the phenotypes belonging to Cellular/Biochemical and Vegetative categories. The phenotypes associated with this group are BiomassYield_G, ExtantLeafNumber1_J, PH-EH_L, PlantHeight_G, and VascularBundleDensity_D.

**Supplementary Fig. S24**. Local Manhattan plot with ±200 kilobases of pleiotropic peak on chromosome 8 from 126,593,834 to 126,796,503 bp. This peak is associated with the phenotypes belonging to Flowering Time and Vegetative categories. The phenotypes associated with this group are Anthesis4_H, Anthesis_A, Anthesis_J, LeafAngle_J, Silking_A, and Silking_J.

**Supplementary Fig. S25**. Local Manhattan plot with ±200 kilobases of pleiotropic peak on chromosome 8 from 135,045,240 to 135,071,381 bp. This peak is associated with the phenotypes belonging to Inflorescence, Root, and Vegetative categories. The phenotypes associated with this group are LowestBranchAngleAuto_P, PlantHeight_J, RootAngle1_O, and RootWidth4_O.

**Supplementary Fig. S26**. Local Manhattan plot with ±200 kilobases of pleiotropic peak on chromosome 8 from 139,924,627 to 139,925,379 bp. This peak is associated with the phenotypes belonging to Flowering Time and Vegetative categories. The phenotypes associated with this group are ExtantLeafNumber1_J, ExtantLeafNumber2_J, PlantHeight_J, and Silking_A.

**Supplementary Table S1**. Widely used maize community association panels.

**Supplementary Table S2**. Trait values for all phenotypes per accession and associated information per accession. Provided as included Excel file.

**Supplementary Table S3**. Phenotypes and associated information. Provided as included Excel file.

**Supplementary Table S4**. The locations of GWAS peaks, the traits associated with each peak, and the genes adjacent to each peak. Provided as included Excel file.

**Supplementary Table S5**. Individual GWAS hits. Provided as included Excel file.

## Abbreviations

BLUPs: best linear unbiased predictions; bp: base pair; DAG: diacylglycerol; exPVPs: expired plant variety patents; GWAS: genome-wide association study; LD: linkage disequilibrium; MAP: Maize Association Panel; MLM: mixed linear model; mRNA: messenger RNA; NCBI: National Center for Biotechnology Information; PCo: principal coordinate; QTL: quantitative trait locus; RMIP: resampling model inclusion probability; RNA-seq: RNA sequencing; SAM: Shoot Apical Meristem; SNP: single-nucleotide polymorphism; SRA: Sequence Read Archive; TAG: triacylglycerol; WiDiv: Wisconsin Diversity Panel.

### Competing Interests

J.C.S. has equity interests in Data2Bio, LLC; Dryland Genetics LLC; and EnGeniousAg LLC. He is a member of the scientific advisory board of GeneSeek and currently serves as a guest editor for *The Plant Cell*. The other authors declare no conflicts of interest.

### Funding

This material is based upon work supported by the US Department of Energy Advanced Research Projects Agency–Energy (ARPA-E) under Award Nos. DE-AR0001064 and DE-AR0001367, the National Science Foundation under grants OIA-1557417 and OIA-1826781, and US Department of Agriculture (USDA)–National Institute of Food and Agriculture (NIFA)under the AI Institute for Resilient Agriculture (Award No. 2021-67021-35329) and the Foundation for Food and Agriculture Research (Award No. 602757). This research was supported in part by the USDA, Agricultural Research Service Project #5030-21000-068-00D. Mention of trade names or commercial products in this publication is solely for the purpose of providing specific information and does not imply recommendation or endorsement by the USDA. The USDA is an equal opportunity provider and employer.

### Authors’ Contributions

R.V.M. and J.C.S. conceived of the study. R.V.M., G.S., M.G., M.C.T., H.J., C.S., L.N., A.M.T., and B.S. designed and carried out experiments to generate data. R.V.M., M.G., and G.S. analyzed the data. R.V.M. and J.C.S. wrote the initial draft of the manuscript. All authors read and approved the final manuscript.

## Supplementary Material

giac080_GIGA-D-22-00083_Original_Submission

giac080_GIGA-D-22-00083_Revision_1

giac080_Response_to_Reviewer_Comments_Original_Submission

giac080_Reviewer_1_Report_Original_Submission Yu Li -- 5/3/2022 Reviewed

giac080_Reviewer_1_Report_Revision_1 Yu Li -- 5/30/2022 Reviewed

giac080_Reviewer_2_Report_Original_Submission Yingjie Xiao, Ph.D. -- 5/3/2022 Reviewed

giac080_Reviewer_2_Report_Revision_1 Yingjie Xiao, Ph.D. -- 6/6/2022 Reviewed

giac080_Reviewer_3_Report_Original_Submission Roberto Pilu -- 5/15/2022 Reviewed

giac080_Supplemental_Files
