# Supplementary material for: Association mapping across a multitude of traits collected in diverse environments in maize
Source: Gigascience. 2022 Aug 23;11:giac080. doi: 10.1093/gigascience/giac080 (PMC9396454; doi:10.1093/gigascience/giac080)
Supplement: giac080_Response_to_Reviewer_Comments_Original_Submission [file giac080_response_to_reviewer_comments_original_submission.pdf]

Author's Response To Reviewer Comments

Close

Reviewer reports:

Reviewer #1: Mural et al. reported a large-scale association analysis based on publicly published genotype and phenotype datasets and a meta-GWAS. This study provides a good example for mining community association panel data and further identifying candidate genes, pleiotropic loci and G x E. Actually, meta-analysis of GWAS has been used in humans and animals. However, I have some major concerns as follows.
1. This study only used three association panels (MAP, SAM, and WiDiv), as I know, some publicly available genotype and phenotype could be obtained for other association panels, for example the association panel including 368 inbred lines (Li et al., 2013, Nat Genetics, 45(1):43-50. doi: 10.1038/ng.2484), which was used widely in GWAS studies in maize. Can other association panels be integrated into this research, which would provide a rich genetic resource for maize research groups.

We agree with the reviewer that the integration of additional panels has the potential to increase the power of massive community GWAS in maize, a point we now touch on in the discussion section of this revised manuscript (Page 10, Right column, Second paragraph).

In the specific case of the AM508 panel from Li et al 2013 (now cited in the introduction (Page 2, Left column, Third paragraph) and discussion (Page 10, Right column, Second paragraph) of our revised manuscript) we faced two challenges. The first is that only a minority of published GWAS papers for this study include phenotype data for the individual lines.

The greater challenge is that the current genetic marker set used for GWAS with the AM508 panel is from an earlier version of the maize genome -- the website from Li et al 2013 was not accessible to us, but based on gene names and publication data we can narrow it down to B73 RefGen_v2 or v_3. Unfortunately this means the genetic markers used for the AM508 GWAS will not share markers/coordinates with the marker set employed in this study which was based on the B73_RefGen_v4 reference genome.

In the future this could be addressed either through reanalysis published resequencing of the AM508 panel, enabling integrated analysis of North American and Chinese maize diversity panels. We now discuss these options in our revised discussions section (Page 10, Right column, Second paragraph).

2. For association analysis, a total of 1014 unique inbred lines and 162 distinct traits from different association panels were used, but these traits were not measured for each of 1041 inbreds. For example, cellular-related traits were mainly measured in the SAM association panel. Hence, association analysis for cellular-related traits were conducted in SAM or 1014 inbreds. If 1014 inbreds were used to perform association analysis for cellular-related traits, how did you analyze the phenotype data? Please describe the method of phenotype data analysis in the Method section.

We apologize for this confusion. For each trait genetic data was subset to only that subset of individuals for which measured trait values were present. We have revised our methods section to make this less ambiguous (Page 12, Left column, Second paragraph of the “Quantitative Genetic Analysis of Trait Data” subsection).

3. Authors used RMIP values to identify significant association signals, please add more details about the RMIP method. What advantages of the resampling-based genome-wide association strategy over other methods?

You are right, this was a significant omission. We have added a new paragraph to the introduction discussing the RMIP method (Page 2, Right Column, Second Paragraph). Briefly, RMIP allows us to employ the FarmCPU algorithm which provides greater power to detect additional trait associated loci which might be masked by the effects of one or more large effect genes without the instability of results

found in single individual runs of the FarmCPU GWAS algorithm. As one of our big goals in conducting this analysis was to generate a dataset that would enable other researchers to compare their results to. For this purpose the stability of marker trait associations is of particular value.

4. Although some important functional genes could be identified, were some new candidate genes obtained in this study functionally verified by the mutants or overexpression experiments.

Unfortunately no, the scope of our study did not permit us to conduct transgenic or gene knock out validation of new candidate genes. We have revised our discussion section to emphasize some of our highest priority candidates for future validation.

5. The authors identified pleiotropic loci based on categories of phenotypes associated with the same peak. For example, the phenotypes associated with the pleiotropic peak on chromosome 8 from 134,706,389 to 134,759,977 bp belongs to Flowering Time, Root and Vegetative categories, thus the locus was associated with different traits. Do you have any ideas on pleiotropic genes based on the results?

We have revised our manuscript to include discussion of both cases where a known causal gene is associated with a pleiotropic locus identified in our study (e.g. MADS69, Liang et al 2018), as well as to discuss in more detail potential candidate genes for two loci with pleiotropic effects without previously known and validated candidate genes associated (Page 10, Right Column, Third Paragraph)

Reviewer #2: The authors described a study of integrating multiple published datasets for reanalysis. They combined previously community panel data and newly collected data in the present study, finally assembling 1014 accessions with 18M SNP markers and 162 traits at different environments. They used a resample-based GWAS method to reanalyze this assemble dataset, and identified 2154 suggestive associations and 697 confident associations. They found genetic loci were pleiotropic to multiple traits. As the authors mentioned, I acknowledge their efforts for collecting and assembling different sources of previously datasets, which should be useful for the maize community. However, to the manuscript per se, I feel the paper seems not to be sufficiently quantified regarding the novelty and significance of reported findings.
If the authors could present several novel results because the previous studies had the limitations on population size, diversity, trait dimensions and environments. In this study, the authors seemed trying to present like this, but it may be improved further and more. It's hard to let me understand there are some novel things which was found due to the merged large dataset. On the other hand, using this assembled dataset, I'm not very clear what's the scientific questions that the authors want to address.

This is a very insightful comment that speaks to the difference approaches research groups take to science. Our goal in assembling this dataset was not to address a specific scientific question, but to generate a dataset/resource which would be valuable and reusable for multiple scientific research avenues. Essentially this boils down the distinction between hypothesis testing research and hypothesis generating research.

In terms of what novel things that were found as a result of our initial proof of concept analyses of this large dataset our intent was to emphasize both the quantitative genetic evidence for pleiotropy between above ground and below ground traits. In this revised manuscript we have revised and added text to emphasize the novelty of this finding. See also our response to reviewer #1 point #4.

In technical sense, I'm wondering how did authors deal with the batch effects when merging datasets phenotype from different environments? It's not comparable for the phenotypes from different accessions collected in different environments. It's hard to figure out the phenotypic difference is caused by genotype, environment, or their interaction.

We apologize that this was not explained sufficiently clear in our previous version. We did not merge data across multiple environments, unless the merging was conducted by the authors prior to making them public by the authors. We have revised our methods section to clarify this that we analyzed these datasets separately rather than merging and producing batch effects (Page 12, Left column, Second paragraph of “Quantitative Genetic Analysis of Trait Data” subsection). This means when a peak is identified in one dataset and not another for same phenotype from different studies is because of genotype by environment interacts that we now discuss in greater length in the discussion section (Page 10, Left Column, Third Paragraph).

The introduction section lacked the proper review for the project background, related progress and publications and findings.

We were sorry to read that the reviewer feels we did not provide sufficient background and citations in the previous version of this manuscript, and assure him or her that it was not our intent to minimize or omit references to the work of other researchers within this field.

While we are unsure what specific publications and findings the reviewer feels were improperly omitted by us, this revised manuscript includes an expanded introduction and discussion and 14 additional new citations not present in the original manuscript.

Reviewer #3: The manuscript "Association Mapping Across a Multitude of Traits Collected in Diverse Environments in Maize" by Ravi V. Mural et al. reported the application of high-density genetic marker data from two partially overlapping maize association panels, comprising 1,014 unique genotypes grown in seven US states, allowing the identification 2,154 suggestive marker-trait associations and 697 confident associations and suggesting the possible application to study gene functions, pleiotropic effects of natural genetic variants and genotype by environment interaction.

The background data are well documented, experimental data are convincing, clearly presented and well discussed, the paper is suitable for publication in Giga Science in its present form.

Thank your for your kind words regarding our manuscript and work.

Close
