# Supplementary material for: Association mapping across a multitude of traits collected in diverse environments in maize
Source: Gigascience. 2022 Aug 23;11:giac080. doi: 10.1093/gigascience/giac080 (PMC9396454; doi:10.1093/gigascience/giac080)
Supplement: giac080_Reviewer_1_Report_Original_Submission — Yu Li -- 5/3/2022 Reviewed [file giac080_reviewer_1_report_original_submission.pdf]

Reviewer Report

Title: Association Mapping Across a Multitude of Traits Collected in Diverse Environments in Maize

Version: Original Submission **Date:** 5/3/2022

Reviewer name: Yu Li

Reviewer Comments to Author:

Mural et al. reported a large-scale association analysis based on publicly published genotype and phenotype datasets and a meta-GWAS. This study provides a good example for mining community association panel data and further identifying candidate genes, pleiotropic loci and G x E. Actually, meta-analysis of GWAS has been used in humans and animals. However, I have some major concerns as follows.

1. This study only used three association panels (MAP, SAM, and WiDiv), as I know, some publicly available genotype and phenotype could be obtained for other association panels, for example the association panel including 368 inbred lines (Li et al., 2013, Nat Genetics, 45(1):43-50. doi: 10.1038/ng.2484), which was used widely in GWAS studies in maize. Can other association panels be integrated into this research, which would provide a rich genetic resource for maize research groups.
2. For association analysis, a total of 1014 unique inbred lines and 162 distinct traits from different association panels were used, but these traits were not measured for each of 1041 inbreds. For example, cellular-related traits were mainly measured in the SAM association panel. Hence, association analysis for cellular-related traits were conducted in SAM or 1014 inbreds. If 1014 inbreds were used to perform association analysis for cellular-related traits, how did you analyze the phenotype data? Please describe the method of phenotype data analysis in the Method section.
3. Authors used RMIP values to identify significant association signals, please add more details about the RMIP method. What advantages of the resampling-based genome-wide association strategy over other methods?
4. Although some important functional genes could be identified, were some new candidate genes obtained in this study functionally verified by the mutants or overexpression experiments.
5. The authors identified pleiotropic loci based on categories of phenotypes associated with the same peak. For example, the phenotypes associated with the pleiotropic peak on chromosome 8 from 134,706,389 to 134,759,977 bp belongs to Flowering Time, Root and Vegetative categories, thus the locus was associated with different traits. Do you have any ideas on pleiotropic genes based on the results?

Methods

Are the methods appropriate to the aims of the study, are they well described, and are necessary controls included? Choose an item.

Conclusions

Are the conclusions adequately supported by the data shown? Choose an item.

Reporting Standards

Does the manuscript adhere to the journal's guidelines on minimum standards of reporting? Choose an item.

Choose an item.

Statistics

Are you able to assess all statistics in the manuscript, including the appropriateness of statistical tests used? Choose an item.

Quality of Written English

Please indicate the quality of language in the manuscript: Choose an item.

Declaration of Competing Interests

Please complete a declaration of competing interests, considering the following questions:

- Have you in the past five years received reimbursements, fees, funding, or salary from an organisation that may in any way gain or lose financially from the publication of this manuscript, either now or in the future?
- Do you hold any stocks or shares in an organisation that may in any way gain or lose financially from the publication of this manuscript, either now or in the future?
- Do you hold or are you currently applying for any patents relating to the content of the manuscript?
- Have you received reimbursements, fees, funding, or salary from an organization that holds or has applied for patents relating to the content of the manuscript?
- Do you have any other financial competing interests?
- Do you have any non-financial competing interests in relation to this paper?

If you can answer no to all of the above, write 'I declare that I have no competing interests' below. If your reply is yes to any, please give details below.

I declare that I have no competing interests.

I agree to the open peer review policy of the journal. I understand that my name will be included on my report to the authors and, if the manuscript is accepted for publication, my named report including any attachments I upload will be posted on the website along with the authors' responses. I agree for my report to be made available under an Open Access Creative Commons CC-BY license (http://creativecommons.org/licenses/by/4.0/). I understand that any comments which I do not wish to

be included in my named report can be included as confidential comments to the editors, which will not be published.

Choose an item.

To further support our reviewers, we have joined with Publons, where you can gain additional credit to further highlight your hard work (see: https://publons.com/journal/530/gigascience). On publication of this paper, your review will be automatically added to Publons, you can then choose whether or not to claim your Publons credit. I understand this statement.

Yes Choose an item.
