# Supplementary material for: Association mapping across a multitude of traits collected in diverse environments in maize
Source: Gigascience. 2022 Aug 23;11:giac080. doi: 10.1093/gigascience/giac080 (PMC9396454; doi:10.1093/gigascience/giac080)
Supplement: giac080_Reviewer_2_Report_Original_Submission — Yingjie Xiao, Ph.D. -- 5/3/2022 Reviewed [file giac080_reviewer_2_report_original_submission.pdf]

Reviewer Report

Title: Association Mapping Across a Multitude of Traits Collected in Diverse Environments in Maize

Version: Original Submission **Date:** 5/3/2022

Reviewer name: Yingjie Xiao, Ph.D.

Reviewer Comments to Author:

The authors described a study of integrating multiple published datasets for reanalysis. They combined previously community panel data and newly collected data in the present study, finally assembling 1014 accessions with 18M SNP markers and 162 traits at different environments. They used a resample-based GWAS method to reanalyze this assemble dataset, and identified 2154 suggestive associations and 697 confident associations. They found genetic loci were pleiotropic to multiple traits. As the authors mentioned, I acknowledge their efforts for collecting and assembling different sources of previously datasets, which should be useful for the maize community. However, to the manuscript per se, I feel the paper seems not to be sufficiently quantified regarding the novelty and significance of reported findings. If the authors could present several novel results because the previous studies had the limitations on population size, diversity, trait dimensions and environments. In this study, the authors seemed trying to present like this, but it may be improved further and more. It's hard to let me understand there are some novel things which was found due to the merged large dataset. On the other hand, using this assembled dataset, I'm not very clear what's the scientific questions that the authors want to address.
In technical sense, I'm wondering how did authors deal with the batch effects when merging datasets phenotype from different environments? It's not comparable for the phenotypes from different accessions collected in different environments. It's hard to figure out the phenotypic difference is caused by genotype, environment, or their interaction.
The introduction section lacked the proper review for the project background, related progress and publications and findings.

Methods

Are the methods appropriate to the aims of the study, are they well described, and are necessary controls included? Choose an item.

Conclusions

Are the conclusions adequately supported by the data shown? Choose an item.

Reporting Standards

Does the manuscript adhere to the journal's guidelines on [minimum standards of reporting?]() Choose an item.

Choose an item.

Statistics

Are you able to assess all statistics in the manuscript, including the appropriateness of statistical tests used? Choose an item.

Quality of Written English

Please indicate the quality of language in the manuscript: Choose an item.

Declaration of Competing Interests

Please complete a declaration of competing interests, considering the following questions:

- Have you in the past five years received reimbursements, fees, funding, or salary from an organisation that may in any way gain or lose financially from the publication of this manuscript, either now or in the future?
- Do you hold any stocks or shares in an organisation that may in any way gain or lose financially from the publication of this manuscript, either now or in the future?
- Do you hold or are you currently applying for any patents relating to the content of the manuscript?
- Have you received reimbursements, fees, funding, or salary from an organization that holds or has applied for patents relating to the content of the manuscript?
- Do you have any other financial competing interests?
- Do you have any non-financial competing interests in relation to this paper?

If you can answer no to all of the above, write 'I declare that I have no competing interests' below. If your reply is yes to any, please give details below.

I declare that I have no competing interests.

I agree to the open peer review policy of the journal. I understand that my name will be included on my report to the authors and, if the manuscript is accepted for publication, my named report including any attachments I upload will be posted on the website along with the authors' responses. I agree for my report to be made available under an Open Access Creative Commons CC-BY license (http://creativecommons.org/licenses/by/4.0/). I understand that any comments which I do not wish to be included in my named report can be included as confidential comments to the editors, which will not be published.

Choose an item.

To further support our reviewers, we have joined with Publons, where you can gain additional credit to further highlight your hard work (see: https://publons.com/journal/530/gigascience). On publication of this paper, your review will be automatically added to Publons, you can then choose whether or not to claim your Publons credit. I understand this statement.

Yes Choose an item.
